# MEGA-GPT: Artificial Intelligence Guidance and Building Analytical Protocols Using MEGA Software

**DOI:** 10.1093/molbev/msaf101

**Published:** 2025-06-06

**Authors:** John B Allard, Sudhir Kumar

**Affiliations:** Institute for Genomics and Evolutionary Medicine, Temple University, Philadelphia, PA 19122, USA; Department of Biology, Temple University, Philadelphia, PA 19122, USA; Institute for Genomics and Evolutionary Medicine, Temple University, Philadelphia, PA 19122, USA; Department of Biology, Temple University, Philadelphia, PA 19122, USA

**Keywords:** MEGA, ChatGPT, RAG, artificial intelligence, phylogeny

## Abstract

Over the past three decades, the Molecular Evolutionary Genetics Analysis (MEGA) software has evolved into a powerful tool with an ever-expanding suite of functionalities. Yet, despite its user-friendly design and widespread adoption by researchers and students, the software's extensive feature set can overwhelm both new and experienced users who are unfamiliar with its latest capabilities. To address this challenge, we developed MEGA-GPT, an AI-driven resource that leverages ChatGPT augmented with retrieval techniques to guide users through MEGA's analytical workflows via natural language queries. By integrating MEGA's help documentation, version-specific articles, and other key publications, MEGA-GPT enhances ChatGPT's standard responses to deliver step-by-step protocols, clarify analytical settings, and recommend optimal workflows. Our evaluations indicate that MEGA-GPT offers significantly improved guidance while minimizing the hallucinations and inaccuracies observed in standard ChatGPT outputs. We propose that such customized, retrieval-augmented query interfaces can substantially enhance the usability of complex scientific computing packages. MEGA-GPT is freely available to all users with a ChatGPT account by accessing the URL https://tinyurl.com/gpt-mega, which is also integrated into MEGA's graphical user interface.

## Introduction

Molecular Evolutionary Genetics Analysis (MEGA) software is widely used for evolutionary analysis in biological research (Kumar 2022). Originally developed to facilitate a few types of molecular evolutionary and phylogenetic analyses, MEGA has evolved into a comprehensive suite of models, methods, and algorithms for small-scale sequence comparisons to whole-genome studies (Caspermeyer 2018; Kumar et al. 2024). MEGA is being applied in diverse fields of research, including virology, bacteriology, general disease studies, plant biology, conservation biology, systematics, developmental evolution, and population genetics​ (Kumar et al. 2004). It is also popular in classroom settings, where it helps students grasp the fundamentals of molecular evolution and phylogenetics (Kumar et al. 2024).

Despite its user-friendly graphical interface, MEGA's extensive feature set can be overwhelming for new users. It can also be challenging for experienced users, especially as new methods and models are added. While various articles and books have provided guidance, printed resources can become outdated due to MEGA's continuous development (Nei and Kumar 2000; Hall 2013; Caspermeyer 2018; Mello 2018; Kumar et al. 2024). Therefore, we have developed MEGA-GPT, a tool that gives users a better understanding of MEGA's features and facilitates the development of protocols for optimal MEGA use.

## Development of MEGA-GPT

MEGA-GPT is built on a retrieval-augmented generation (RAG) framework, which combines dynamic information retrieval with language generation (Lewis et al. 2020). It takes a hybrid approach, which is particularly effective for domain-specific applications, as it enables large language models to access up-to-date, specialized information beyond their static training data (Lewis et al. 2020). RAG reduces well-known issues such as hallucination and domain insensitivity related to using artificial intelligence (Lewis et al. 2020).

The RAG architecture comprises two key components: a retriever and a generator. The retriever scans indexed databases containing domain-specific content, from scientific articles to comprehensive technical documentation, to extract information relevant to the user's query. Content is retrieved in a vector representation and passed to the generator that synthesizes a response by merging the user's query vectorized representations with this authoritative data. This two-step process enhances factual accuracy by grounding responses in current and specialized sources (Guu et al. 2020; Borgeaud et al. 2022) and reduces errors. Moreover, the RAG framework allows for continuous updates to the knowledge base without requiring extensive model retraining (Lewis et al. 2020; Shuster et al. 2021). This adaptability is crucial in rapidly evolving scientific fields and for software under continuous development, ensuring that MEGA-GPT delivers guidance that reflects the latest methodologies included in MEGA.

MEGA-GPT is based on the custom GPT framework provided by OpenAI et al. (2023), which can be accessed through the ChatGPT interface. In the first version of MEGA-GPT, we have included MEGA help documentation, articles covering various MEGA versions since 1993, and some key articles related to MEGA and methods included therein (Kumar et al. 1994, 2001, 2008, 2012, 2018, 2024; Nei and Kumar 2000; Tamura et al. 2007, 2011; 2013, 2018, 2021; Stecher et al. 2014; Caspermeyer 2018; Mello 2018; Stecher et al. 2020; Tao et al. 2020; Sharma and Kumar 2024).

## Example Use Cases

To evaluate the effectiveness of MEGA-GPT compared to the basic version of ChatGPT (GPT-4o, OpenAI et al. 2024), we posed queries related to MEGA's functionality and assessed the usefulness of the responses. The GPT-4o did very well in many queries, but we found cases where MEGA-GPT performed much better. We describe three examples below.

### Task 1: Diagnosing the Pathogenicity of a Protein Variant

**Fig. 1.**
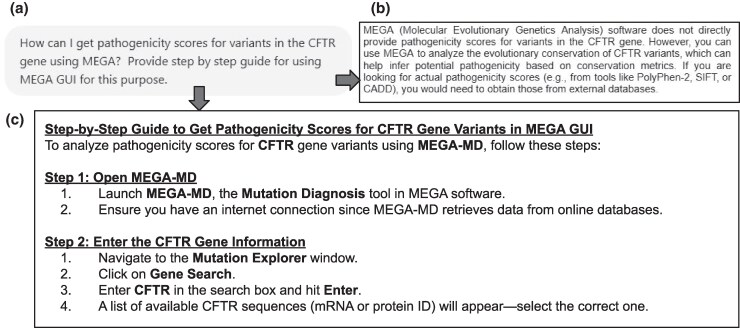
Comparison of outputs for Task 1. a) The query given to both models. b) An excerpt from the response given by GPT-4o which incorrectly claimed that MEGA cannot compute pathogenicity scores. c) Response from MEGA-GPT. Supplementary fig. S1, Supplementary Material online gives additional information provided by MEGA-GPT.

Our query was: “How can I get pathogenicity scores for variants in the CFTR gene using MEGA?” (Fig. 1a). GPT-4o incorrectly responded that MEGA lacks any functionality to provide pathogenicity scores (Fig. 1b). In contrast, MEGA-GPT correctly identified a dedicated functionality (mutational diagnosis) for predicting the functional impact of nonsynonymous single nucleotide variants within MEGA (Stecher et al. 2014) (Fig. 1c). Moreover, MEGA-GPT offered a good step-by-step guide on how to obtain pathogenicity scores for a CFTR variant, explaining that the tool integrates PolyPhen-2, SIFT, and EvoD for mutation impact assessment. It also suggested that MEGA produces CADD scores, but that was a minor inaccuracy. This comparison highlights a key limitation of general-purpose AI assistants like GPT-4o: They may overlook critical features of specialized scientific software, potentially leading to misinformation that could misguide researchers.

### Task 2: Building a Timetree

**Fig. 2.**
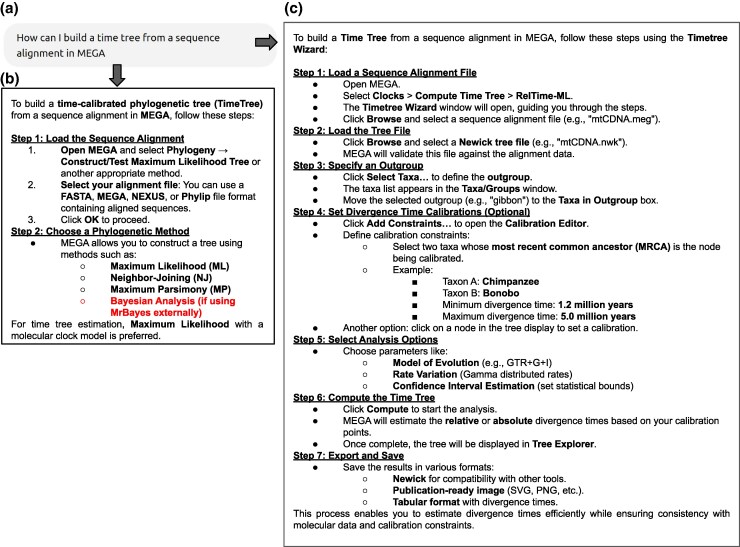
Comparison of outputs for Task 2. a) The query given to both models. b) An excerpt from the response given by GPT-4o which failed to mention the RelTime method and gave incorrect information (problematic text in red that refers to Bayesian Analysis in the text). c) The response given by MEGA-GPT which gave a correct protocol. Full response for panel b) is provided in supplementary fig. S2, Supplementary Material online.

MEGA-GPT's response also outperforms GPT-4o's on common tasks such as generating a timetree. When asked, “How can I build a timetree from a sequence alignment in MEGA?” (Fig. 2a). GPT-4o provided an answer that omitted critical features and introduced significant inaccuracies (Fig. 2b). Notably, it failed to mention MEGA's built-in RelTime method for estimating divergence times (Tamura et al. 2018). Instead, it misleadingly recommended using BEAST, an independent software environment. It also suggested incorrect steps, such as using the nonexistent “Test Molecular Clock” option under the *Phylogeny* menu. It even stated that “Bayesian analysis” could be used to build a timetree in MEGA by linking to MrBayes, another external software tool unrelated to MEGA. These errors illustrate a broader issue of hallucination, where GPT-4o fabricates nonexistent workflow steps, potentially leading users astray and frustrating them when they cannot find those features.

In contrast, MEGA-GPT provided a detailed and accurate protocol using MEGA's Timetree Wizard (Fig. 2c). It correctly outlined the workflow, which involves selecting the alignment file, loading a Newick tree, specifying an outgroup, applying divergence time calibrations, and running the RelTime method to estimate divergence times. Therefore, the RAG technology enabled the responses to focus on MEGA's features. MEGA-GPT demonstrates a clear advantage in delivering accurate, application-specific guidance.

### Task 3: Testing for Recombination

An essential function of an effective AI assistant is accurately communicating the limitations of the software it supports. Users may sometimes inquire about functionalities MEGA does not offer, and the assistant must convey these constraints to avoid confusion and frustration. To assess this, we asked MEGA-GPT and GPT-4o, “How do I use MEGA to test for recombination in a nucleotide alignment?”, a function for which MEGA does not include a built-in tool. GPT-4o fabricated a protocol for this nonexistent feature, claiming that MEGA offers a “Recombination Identification Program (RIP)” and even provided plausible-sounding but false menu options (supplementary fig. S3a, Supplementary Material online). This example highlights a common pitfall of general-purpose AI assistants: the tendency to hallucinate credible yet inaccurate information.

In contrast, MEGA-GPT correctly stated, “MEGA does not have a direct built-in recombination detection tool.” While it mentioned some alternative tests that MEGA can perform, it avoided introducing misleading or fabricated protocols (supplementary fig. S3b, Supplementary Material online). This accurate acknowledgment of MEGA's limitations underscores the value of a tailored, retrieval-augmented generation-based assistant in providing reliable, domain-specific guidance.

### Task 4: Beyond Building Protocols

MEGA-GPT is more than just a protocol provider; it serves as a versatile assistant for MEGA users, offering detailed and use-case-tailored explanations, troubleshooting guidance, and strategic recommendations tailored to specific research needs. Users can inquire about MEGA's capabilities, including available phylogenetic methods, comparisons of different evolutionary models, and supported sequence formats. For example, suppose a user is unsure which substitution model to apply. In that case, MEGA-GPT can explain the differences, such as when to choose the Tamura-Nei (1993) model over the Jukes-Cantor (1969) model or how Gamma-distributed rate variation impacts branch length estimates.

In addition to setting up analyses, MEGA-GPT assists with interpreting results. If a user encounters unexpected bootstrap support values or discrepancies in divergence time estimates, MEGA-GPT can help diagnose potential issues, such as missing calibration points or unsuitable outgroup selection. For researchers working with large datasets, it also suggests strategies to optimize computational efficiency, guides file format conversions, troubleshoots common errors, and summarizes new features and improvements in the latest version of MEGA. While general-purpose AI addresses some of these questions, MEGA-GPT's specialized focus on MEGA's functionalities ensures more accurate and context-specific guidance.

### Conclusion and Future Directions

By integrating artificial intelligence, MEGA-GPT enhances the accessibility of MEGA for users ranging from novices to seasoned bioinformaticians. It represents another step toward making computational tools for evolutionary genetics more accessible. This will lower barriers to entry, improve efficiency, and reduce user error, empowering new and experienced users to harness MEGA's advanced capabilities more fully. By enabling users to engage in a dialog with the help documentation, which is otherwise static, MEGA-GPT allows users to seek clarification and ask follow-up questions. These features advance beyond the traditional paradigm, where text searches and lists of frequently asked questions are automated by MEGA-GPT. This also significantly enhances MEGA's role in education settings, helping to train future bioinformaticians. MEGA-GPT is accessible from the MEGA GUI alongside traditional help docs, making it readily available as an AI assistant that leverages retrieval-augmented generation to deliver better, context-aware responses.

With the development of MEGA-GPT, we see a shift in the development of scientific software. Many advanced computational tools can be intimidating without significant training, limiting their adoption and impact. AI-powered assistants, specifically tailored to domain-relevant knowledge, offer a promising solution to bridge this gap, especially as tools like ChatGPT become more widespread across the sciences and in the general population. While MEGA-GPT is an early forerunner in our field, we expect many more such assistants to become available soon.

## Supplementary Material

msaf101_Supplementary_Data

## Data Availability

MEGA-GPT is freely available to all users with a free or paid ChatGPT account at https://chatgpt.com/g/g-RmeN18Ssp-mega-gpt and https://tinyurl.com/gpt-mega. It is also accessible through the MEGA GUI interface, in the *Help* menus, and the main MEGA window.
