# Supplementary material for: MEGA-GPT: Artificial Intelligence Guidance and Building Analytical Protocols Using MEGA Software
Source: Mol Biol Evol. 2025 Jun 6;42(6):msaf101. doi: 10.1093/molbev/msaf101 (PMC12141744; doi:10.1093/molbev/msaf101)
Supplement: msaf101_Supplementary_Data [file msaf101_supplementary_data.pdf]

Supplementary Figures for

Supplementary Figure S1. Complete response from MEGA-GUI for Task 1.

Step-by-Step Guide to Get Pathogenicity Scores for CFTR Gene Variants in MEGA GUI
To analyze pathogenicity scores for **CFTR** gene variants using **MEGA-MD**, follow these steps:

Step 1: Open MEGA-MD

1. Launch **MEGA-MD**, the **Mutation Diagnosis** tool in MEGA software.
2. Ensure you have an internet connection since MEGA-MD retrieves data from online databases.

Step 2: Enter the CFTR Gene Information

1. Navigate to the **Mutation Explorer** window.
2. Click on **Gene Search**.
3. Enter **CFTR** in the search box and hit **Enter**.
4. A list of available CFTR sequences (mRNA or protein ID) will appear—select the correct one.

Step 3: Specify the Variants
You can specify variants in two ways:
Option 1: Manually Enter Variants

- Click on the **Sequence Data Explorer**.
- Navigate to the amino acid position of interest.
- Select the **mutant allele** (e.g., F508del, G551D).
- Click **Diagnose Variant**.

Option 2: Upload a Variant List
Create a **text file** with variant details in the format:

```
NP_000492  508  F
NP_000492  551  G
```

- Here, NP_000492 is the **RefSeq protein ID**, 508 is the **amino acid position**, and F is the **mutant allele**.
- Go to **File → Import Query Data From File** and select your prepared file.
- MEGA-MD will validate the file and retrieve results.

Step 4: Retrieve Pathogenicity Scores

1. MEGA-MD queries the **MEGA-MDW** (a web-based variant diagnosis server).
2. The results will be displayed in the **Predictions Tab** with:
    - **Mutation** details
    - **Predicted impact score** (PolyPhen-2, SIFT, EvoD)
    - **Evolutionary context**
3. You can sort, search, and export results using the provided options.

Step 5: Explore Ancestral Information (Optional)

- Click **Explore Ancestors** to infer ancestral states of the CFTR variant.
- Choose **Maximum Likelihood (ML)** or **Maximum Parsimony (MP)** to estimate its evolutionary history.

Step 6: Save and Export Results

- Click **Export Table to CSV** or **Export Table to Excel** to save your analysis.
- You can also generate a **detailed view** for each variant.

Step 7: (Alternative) Use the MEGA-MDW Web Interface
If analyzing many variants, use **MEGA-MDW** directly:

1. Go to **www.mypeg.info/evod**.
2. Upload the same **variant list** file.
3. Wait for an email with the pathogenicity scores.

Supplementary Figure S2. Complete response from ChatGPT-4o for Task 2.

To build a **time-calibrated phylogenetic tree (TimeTree)** from a sequence alignment in **MEGA**, follow these steps:

<u>Step 1: Load the Sequence Alignment</u>

1. **Open MEGA** and select **Phylogeny → Construct/Test Maximum Likelihood Tree** or another appropriate method.
2. **Select your alignment file**: You can use a **FASTA**, **MEGA**, **NEXUS**, or **Phylip** file format containing aligned sequences.
3. Click **OK** to proceed.

<u>Step 2: Choose a Phylogenetic Method</u>

- MEGA allows you to construct a tree using methods such as:
    - **Maximum Likelihood (ML)**
    - **Neighbor-Joining (NJ)**
    - **Maximum Parsimony (MP)**
    - **Bayesian Analysis (if using MrBayes externally)**

For time tree estimation, **Maximum Likelihood** with a molecular clock model is preferred.

<u>Step 3: Convert the Tree to a TimeTree</u>

1. **Enforce a Molecular Clock**:
    - After constructing the tree, select **"Test Molecular Clock"** under the **Phylogeny** menu.
    - If the clock is rejected, you may need to use a relaxed clock method in external software like BEAST.
2. **Calibrate the Tree**:
    - Select **"Compute TimeTree"** in MEGA.
    - Specify **calibration points**, which can be based on **fossil records** or **secondary calibrations** (e.g., divergence times from prior literature).
    - If available, enter **known divergence times** for one or more nodes.
3. **Run the TimeTree Analysis**:
    - Choose an **evolutionary model** (e.g., **Tamura-Nei, GTR**).
    - Adjust the **rate variation model** (e.g., Gamma distribution).
    - Click **Compute** to generate a time tree.

<u>Step 4: Interpret and Save the Tree</u>

- Once the tree is computed:
    - View branch lengths **scaled to time**.
    - Export the tree in **Newick** or **MEGA format** for further analysis.
    - Visualize divergence times and confidence intervals.

Alternative: Use MEGA for Sequence Alignment, Export to BEAST for TimeTree
If you need more advanced time-tree models (e.g., relaxed clock), consider:

1. **Align sequences in MEGA**.
2. **Export the alignment** in **Nexus format**.
3. **Use BEAST2** to construct a Bayesian TimeTree.
4. Would you like detailed steps on using **MEGA with BEAST** for better time calibration?

Supplementary Figure S3. Comparison of outputs for Task 3. (a) The query given to both models. (b) An excerpt from the response given by GPT-4o, which fabricated an imaginary tool called "Recombination Identification Program," and an imaginary protocol to go with it. Incorrect text is highlighted in red. (c) The response given by MEGA-GPT correctly notes that MEGA does not have a built-in tool to test for recombination (highlighted in blue), but it suggests some tests that MEGA does provide that may be of interest to the user.

a

How do I use MEGA to test for recombination in a nucleotide alignment

b

To test for recombination in a nucleotide alignment using MEGA, follow these steps:

Step 1: Prepare Your Alignment

- Ensure that your sequences are **properly aligned**. You can use MEGA's **Alignment Explorer** to align sequences using ClustalW or MUSCLE.
- Save your alignment in MEGA format (`.meg`) or another supported format such as FASTA.

Step 2: Perform Recombination Analysis

MEGA does not have a direct built-in recombination detection tool, but it provides **tests of selection and neutrality**, which can indicate potential recombination events. You can use Tajima's D test and related neutrality tests:

...

c

You can use MEGA (Molecular Evolutionary Genetics Analysis) to test for recombination in a nucleotide alignment using **RIP (Recombination Identification Program)**, which is included in MEGA. Here's how you can do it:

Steps to Test for Recombination in MEGA

1. **Open MEGA and Load Your Alignment**
   - Launch **MEGA** and go to **File** → **Open a File/Session**.
   - Select your **nucleotide alignment** file (in **FASTA, MEGA, or Phylip** format).
   - Ensure the sequences are aligned properly before proceeding.
2. **Run the Recombination Detection Test (RIP)**
   - Go to **Tools** → **Recombination Detection (RIP)**.
   - In the **RIP Analysis Setup**, you will see options to define the window size, step size, and other parameters.
   - Adjust the **window size** (e.g., 200 bp) and **step size** (e.g., 20 bp) based on your alignment length.
   - Choose a **substitution model** for the analysis (e.g., Tamura-Nei).
3. **Run the Analysis**
   - Click **Compute** to start the recombination detection analysis.
   - MEGA will scan for recombination breakpoints across the alignment.
4. **Interpret the Results**
   - The results will show a **graphical representation** of recombination signals across the alignment.
   - Look for regions where genetic distance patterns shift abruptly, indicating potential recombination breakpoints.
   - If MEGA detects significant recombination events, it will suggest potential breakpoints...
